# A benchmark survey of plankton, fish and benthic composition in Poblacion and Kadurong Reefs in Liloan, Cebu, Philippines

**DOI:** 10.3897/BDJ.9.e72537

**Published:** 2021-09-23

**Authors:** Brisneve Edullantes, Fleurdeliz Maglangit, Angelito M. Ortiz, Joana Mie R. Casibo, Lorraine Louise C. Vicentuan, Eukene O. Bensig

**Affiliations:** 1 Department of Biology and Environmental Science, College of Science, University of the Philippines Cebu, Cebu City, Philippines Department of Biology and Environmental Science, College of Science, University of the Philippines Cebu Cebu City Philippines

**Keywords:** coral reef, reef fish, zooplankton, phytoplankton, water quality, marine protected area, MPA

## Abstract

**Background:**

Coral reefs offer valuable ecosystem goods and services, such as coastal protection, erosion regulation, fishery, biodiversity, habitat and nursery grounds. However, they face threats from anthropogenic activities, including poor water quality, global warming, coastal development and unsustainable fisheries. Marine Protected Areas (MPAs) provide a structured and holistic approach in addressing these threats. Regular monitoring and assessment of these MPAs are crucial components in evaluating the MPAs design and effectiveness. Two coral reefs (i.e. Poblacion and Kadurong Reefs) were established as MPAs in Liloan, Cebu, Philippines to protect crucial habitat and biodiversity with the hope of improving fisheries by avoiding fish stock disintegration. These coral reefs provide shelter to many commercially-significant fish species, supporting subsistence and livelihood in the community. These MPAs are not only biologically rich, but they also support socio-economic stability. Hence, management and protection of the coral reefs in the MPAs of Liloan, Cebu is of paramount importance. To formulate conservation and applicable management measures, research and monitoring should be in place. This paper presents the data collected from the short term monitoring in the Poblaction and Kadurong Reefs. The paper describes an important set of data that can be used by the stakeholders to benchmark biophysical assessments for management of marine-protected areas in Liloan.

**New information:**

This data paper provides baseline information on the health of the coral reefs of the MPAs in Liloan, Cebu. Datasets covering physico-chemical and biological parameters inclusive of water quality, coral reef cover, fish and plankton occurrence and abundance were determined using the standard protocols for surveying tropical marine resources. The results will serve as a benchmark in formulating guidelines and implementing relevant policies for the effective management and protection of the MPAs in Liloan, Cebu, Philippines.

## Introduction

Coral reefs are considered to be the most biodiverse habitat on earth (Messmer et al. 2011). They accommodate a variety of species that maintain the balance of the marine ecosystem. They are nurseries to juvenile marine organisms and support over 25% of fishes in the ocean and up to two million marine species. They protect the shores from the impacts of waves, provide food and medicine to humans and are economically important to local communities and tourism (Moberg and Folke 1999).

However, these marine ecosystems are facing a wide variety of threats, ranging from natural and anthropogenic activities, such as poor water quality, global warming, coastal development and unsustainable fisheries (Mumby and Steneck 2010, Schaffelke et al. 2005, Wenger et al. 2015). Marine Protected Areas (MPAs) provide a structured and holistic approach in addressing these threats and are one of the most promising solutions to increase resilience of coral reefs (Cinner et al. 2016). MPAs are specified areas of coastal land and water that are defined to protect natural resources and ecosystems and to halt the decline of marine biodiversity (Zhao et al. 2020). They have been established as an alternative approach in lieu of traditional practices not only in conserving marine biodiversity, but also in managing fisheries sustainability (Mumby and Steneck 2010). In the Philippines, MPAs have been established since the 1970s and more than 500 MPAs (104,176 hectares) were legally established all over the country (Garces et al. 2013). However, only a few percent (below 5%) of the coral reefs are currently protected in the Philippines (Weeks et al. 2010). Despite the many potential benefits of establishing MPAs in the coastal areas, the majority of the MPAs in the country do not meet their management objectives. Regular monitoring and assessment of MPAs can contribute to formulate relevant options for conservation and protection management of MPAs (Wilkinson et al. 2003, Cooper et al. 2009, Fabricius et al. 2014).

This data paper presents the sampling-event dataset of the short-term monitoring in Poblacion and Kadurong Reefs, two of the marine protected areas in the Municipality of Liloan, Cebu, Philippines. Water quality and ecological assessments were carried out to monitor the status and trends of biological and physical parameters associated with coral reefs using the standard protocols for surveying tropical marine resources. Specifically, the following measurements were conducted: (1) physico-chemical parameters, (2) phytoplankton and zooplankton occurrence and abundance, (3) fish occurrence and density and (4) percent cover of benthic components of coral reef. Aside from coral reef and fish assessment, the coastal waters of Liloan require monitoring of plankton community structure and physico-chemical parameters because of the significance of phytoplankton and zooplankton community response to environmental variables for interpreting ecological variations amid threats of anthropogenic activities, such as climate change and pollution. The data can serve as the basis for the formulation and implementation of relevant measures for conservation and protection management of the Poblacion and Kadurong Reefs in Liloan, Cebu, Philippines (Edullantes et al. 2021, Flores et al. 2020, Murphy et al. 2020).

## Project description

### Personnel

The surveys were mainly conducted by the authors, either as individuals or as groups, with the help of volunteers and experienced marine biologists. Brisneve Edullantes supervised the phytoplankton and zooplankton surveys. Eukene Bensig supervised the coral reef and fish monitoring. Fleurdeliz Maglangit supervised the water quality assessment. The surveys were conducted in coordination with the Local Government Unit (LGU) in the Municipality of Liloan, Cebu, Philippines.

### Study area description

**Figure 1.**
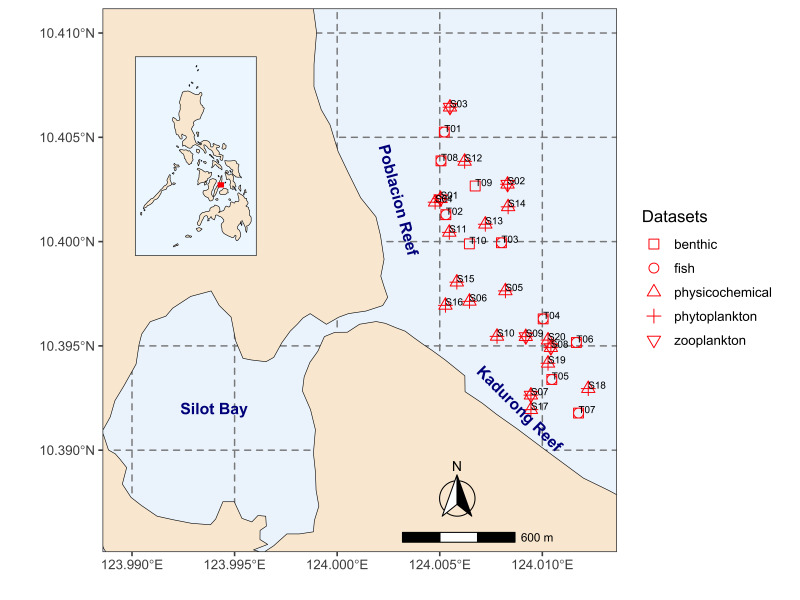
Map of the study area and location of sampling sites in the Poblacion and Kadurong Reefs of Liloan, Cebu, Philippines.

Poblacion and Kadurong Reefs are located in Barangay Poblacion, Municipality of Liloan, Cebu, Philippines (Fig. 1). The MPAs in Poblacion and Kadurong cover 16.81 and 4.76 hectares, respectively. These reefs are adjacent to Silot Bay, a semi-enclosed bay connected to the coastal waters via a narrow inlet with several eddies. The shores near the reefs are lined with residential and commercial areas, including a shipyard on the inlet connecting the Silot Bay and the semi-enclosed bay.

### Design description

Water quality and diversity assessments were carried out to assess the status and trends of biological and physical parameters associated with coral reefs using the standard protocols for surveying tropical marine resources.

### Funding

This study was funded by the University of the Philippines Cebu Creative Work and Research Grant.

## Sampling methods

### Sampling description

A total of 30 sampling locations were selected for the study (Fig. 1). Twenty (20) of the locations were sampling sites for the physico-chemical assessment and phytoplankton survey (S01-S20) and for zooplankton survey (S01-S03 and S07-S09). Ten (10) of the locations were transects for the coral reef and fish assessments (T01-T10). Different measurements were conducted in these sampling locations in March 2015, 2016 and July 2016.

**Figure 2.**
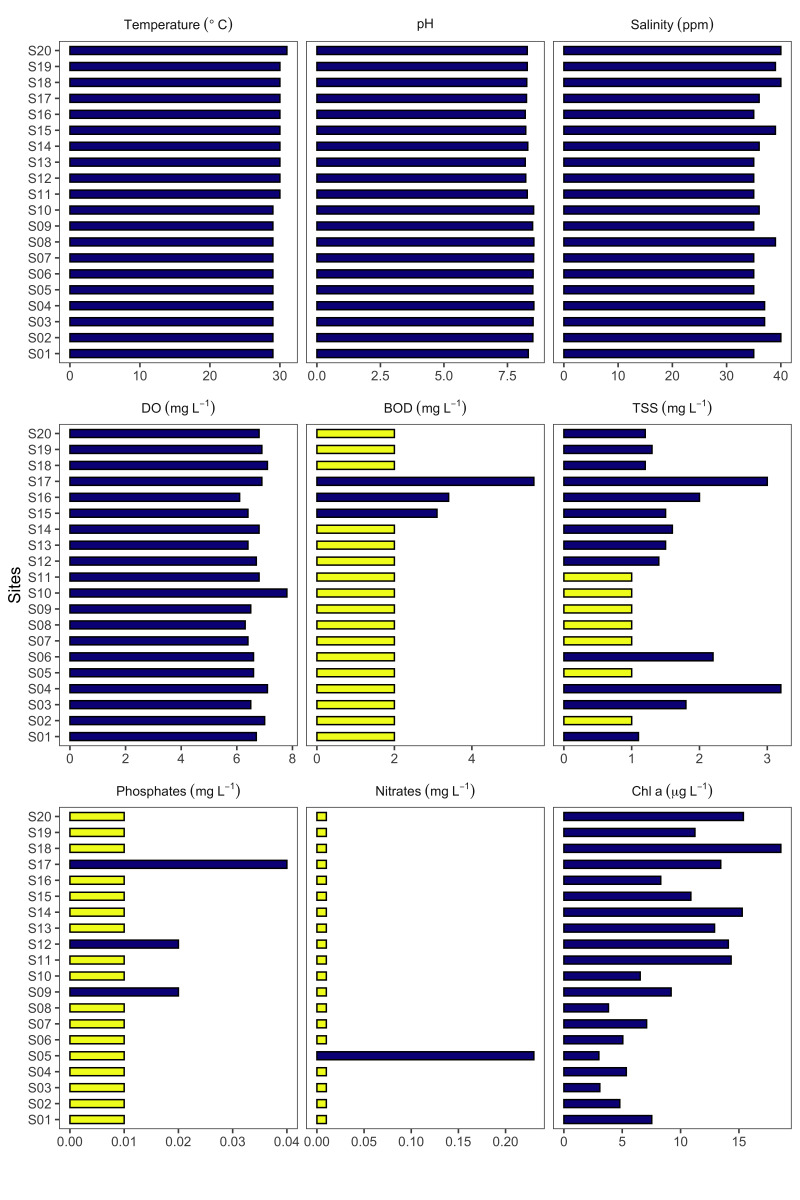
Mean values of physico-chemical parameters recorded at the different sampling sites in Poblacion and Kadurong Reefs. Yellow bars indicate that the measurements were below the detection limit (i.e. BOD = 2 mg l^-1^, TSS = 1 mg l^-1^, phosphates and nitrates = 0.01 mg l^-1^).

**Physico-chemical measurements**: Physico-chemical measurements were conducted at all sampling sites (S01-S20) in March 2015 and 2016. The following physico-chemical parameters were measured in situ for each of the sampling sites (S01-S20): temperature (°C) using a calibrated thermometer, pH with a standard portable pH meter (Mettler Toledo) and salinity (ppm) using a refractometer (Fisherbrand™ handheld analogue salinity refractometer). All in situ parameters were measured in triplicate. Water samples were collected by grab sampling for the analysis of Dissolved Oxygen (DO, mg l^-1^), Biochemical Oxygen Demand (BOD, mg l^-1^), Total Suspended Solids (TSS, mg l^-1^), total phosphates (mg l^-1^) and nitrates (mg l^-1^). All sampling bottles were acid-washed, cleaned, rinsed with distilled water and dried before use. Collected water samples were stored in an ice bucket (4°C) and transported to the laboratory for analysis. The samples were kept at this temperature (4°C) for 24 h if treatment was not immediate. All the analyses were performed in triplicate as described previously (Bensig et al. 2014, Maglangit et al. 2014, Maglangit et al. 2016) following the standard protocols in APHA (Eaton et al. 1995). In brief, the DO was determined by azide modification (Winkler) method, BOD by azide modification (dilution) technique, TSS by gravimetric method, total phosphates by chromotropic-colourimetric method and nitrates by stannous chloride reduction method. For analysis of chlorophyll, a concentration, another 1 litre surface seawater was taken in each sampling point and was processed immediately in the laboratory. About 500 ml of water sample was filtered using a Whatman GF/C filter. The filtered phytoplankton sample was extracted in 8 ml 90% acetone for 24 hours. Chlorophyll a concentration (Chl a, μg l^-1^) was estimated spectrophotometrically following the standard protocol (Eaton et al. 1995) with three replicates. Mean values of the physico-chemical parameters were reported in emof.csv dataset. The data are visualised in Fig. 2.

**Figure 3.**
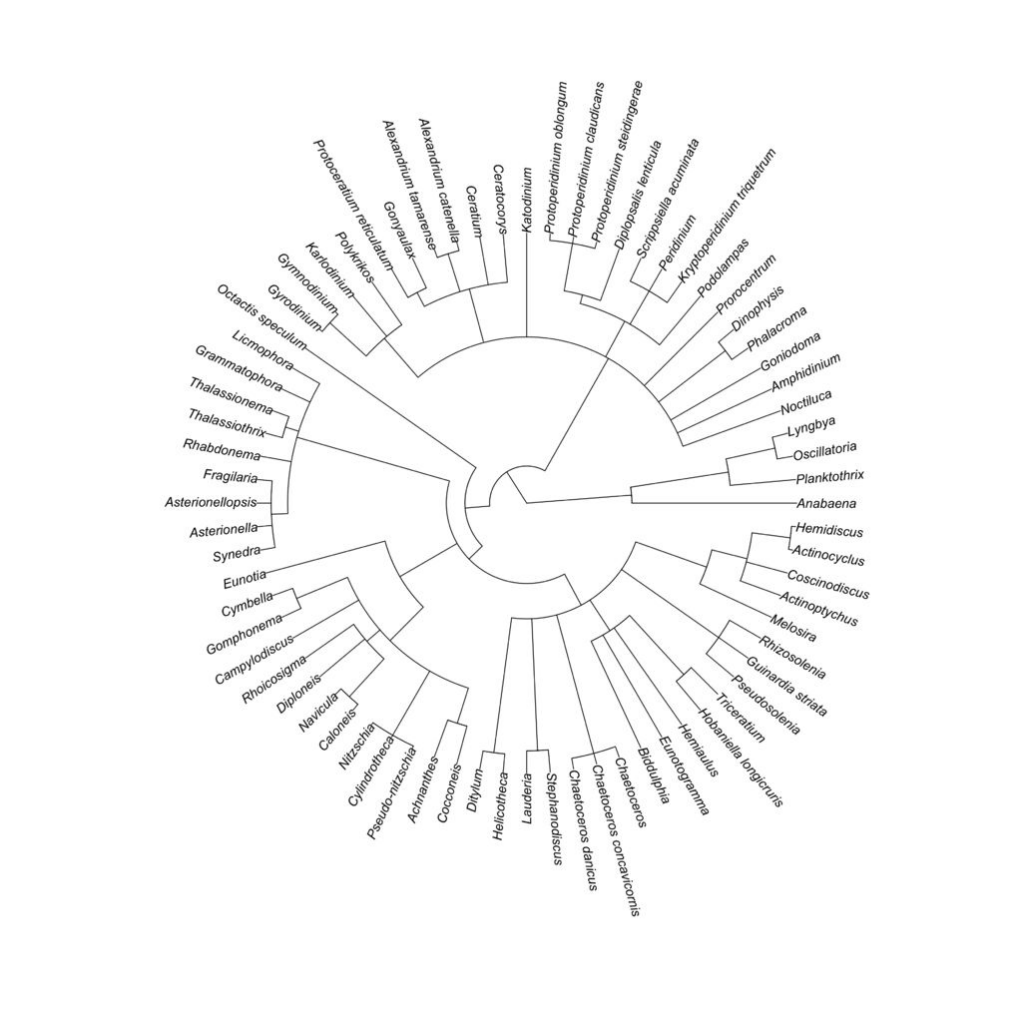
Taxonomic tree of the phytoplankton occurring in Kadurong and Poblacion Reefs.

**Figure 4.**
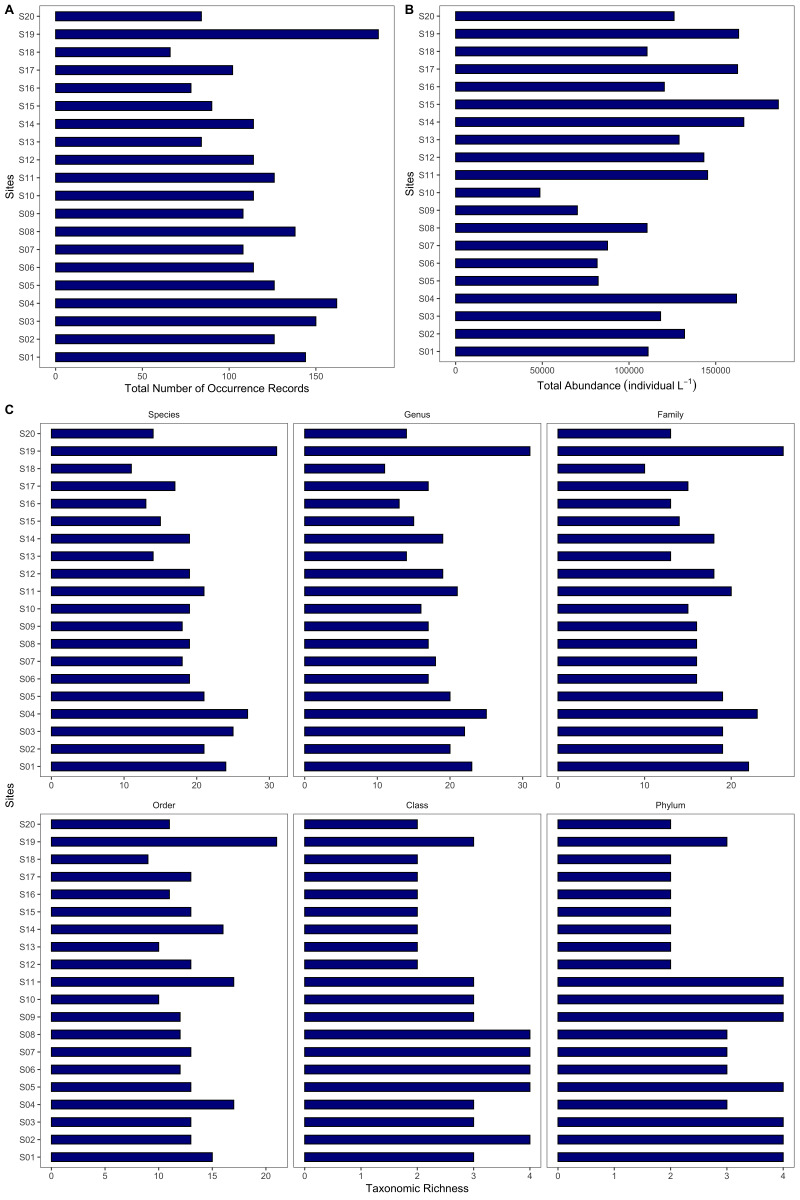
Overview of the phytoplankton dataset: **A** total number of occurrence records of phytoplankton across sites; **B** total abundance of phytoplankton across sites and **C** total number of species, genus, family, order, class and phylum (taxonomic richness) of phytoplankton across sites.

**Phytoplankton assessment**: Phytoplankton samples were collected in each of the sampling sites (S01-S20). Fifteen (15) litres of surface seawater were collected 0.5 m below the surface. The collected water sample will be subsequently sieved with a 20 μM mesh phytoplankton net and was stored in a 1 litre polyethylene bottle preserved with roughly 5 ml Lugol’s Solution. The seawater samples were labelled accordingly and were brought to the laboratory for analysis. The samples were allowed to stand for 48 hours. Thereafter, the upper portion was decanted leaving 100 ml of concentrated phytoplankton sample. The sample was gently homogenised before a 1 ml aliquot was pipetted out for microscopy. One to two drops of the 1 ml aliquot was examined under the microscope under 10× and 40× magnification at a time using the drop-count method (Verlencar et al. 2004). Phytoplankton individuals were counted and photographed. Phytoplankton were identified at the lowest taxonomic level possible. In the case of colonial and filamentous phytoplankton, filaments and colonies were considered individual phytoplankton. The phytoplankton found were verified in the WoRMS database (WoRMS Editorial Board 2015). The database returns information about the taxonomic classification of genera sent. Abundance per sampling site was computed using the following formula: abundance = (I x A)/l, where I is equal to the number of phytoplankton individuals found per 1 ml aliquot (individual ml^-1^), A is equal to the 100 ml concentrated phytoplankton sample and l is equal to the total amount of seawater sieved in the phytoplankton net which is 15 l. Occurrence and abundance (individual l^-1^) of phytoplankton were reported in the occurrence.csv dataset. The data are visualised in Fig. 3 and Fig. 4.

**Figure 5.**
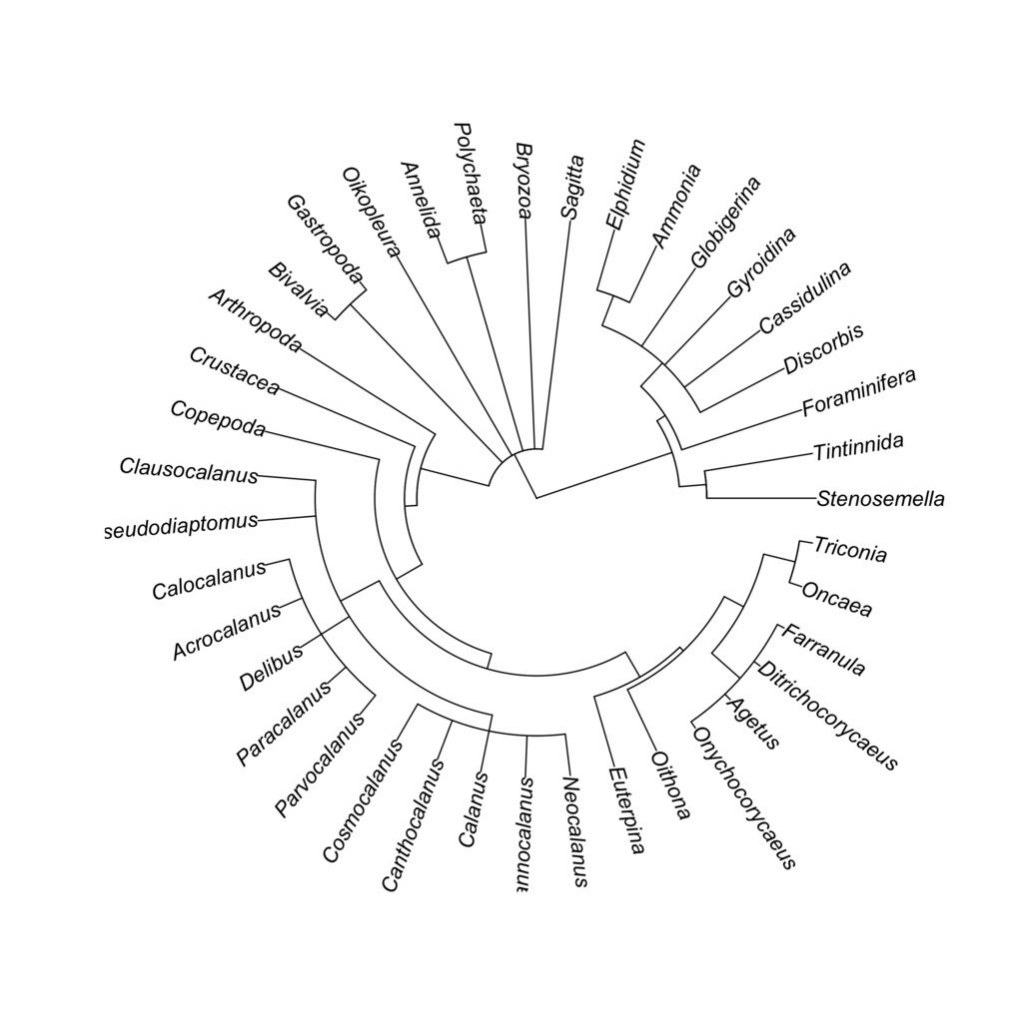
Taxonomic tree of the zooplankton occurring in Kadurong and Poblacion Reefs.

**Figure 6.**
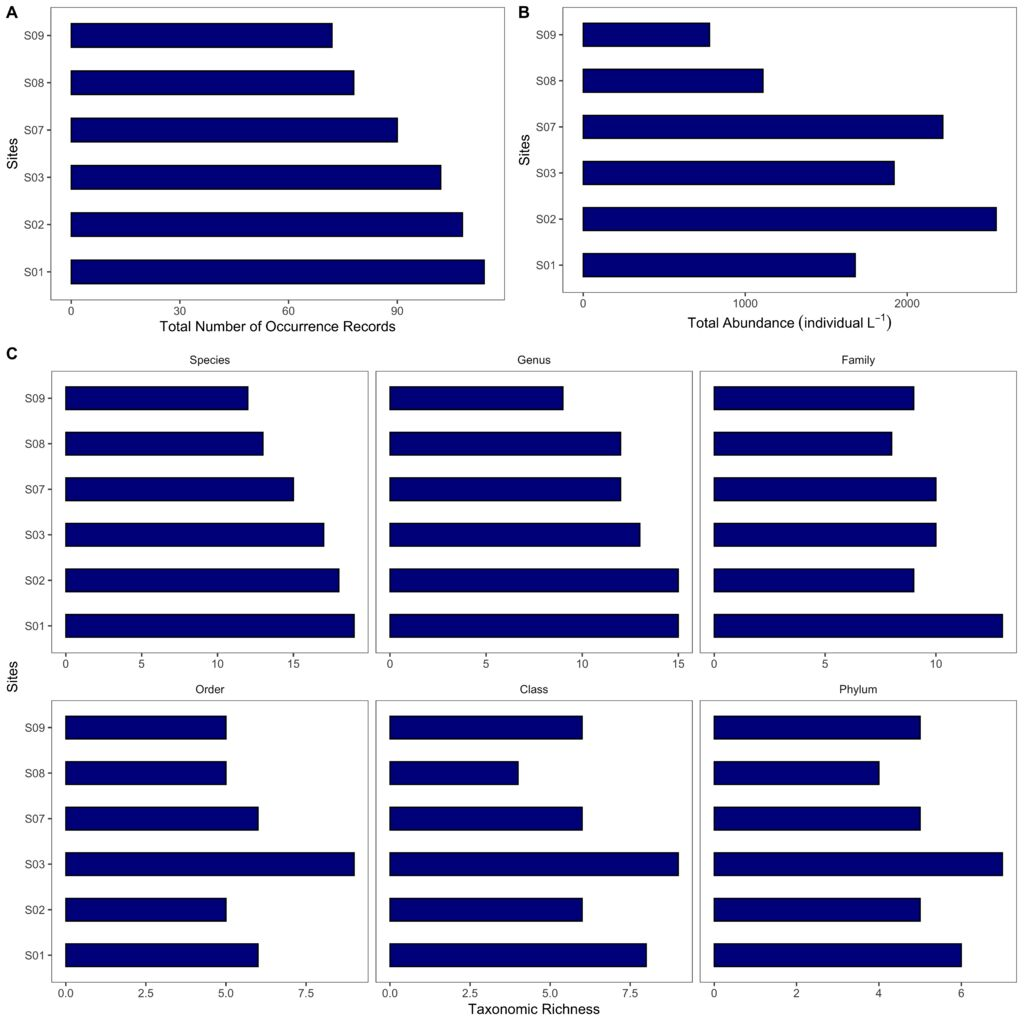
Overview of the zooplankton dataset: **A** total number of occurrence records of zooplankton across sites; **B** total abundance of zooplankton across sites and **C** total number of species, genus, family, order, class and phylum (taxonomic richness) of zooplankton across sites.

**Zooplankton assessment**: Composite sampling was employed for zooplankton; thus there were three points per sampling site (S01-S03 and S07-S09). Collection of zooplankton was done using the standard mesh net with a stopcock at the lower end to allow opening and closing. A calibrated dipper was used to obtain water samples at approximately 0.1-0.5 m from the surface. The collected water sample was passed through the mesh net (stopcock closed) to allow sieving of zooplankton. This provided a more concentrated number of species. Total water sample passed through the net was 30 l. The stopcock was opened when the last few millilitres of water sample were passed through the mesh net, with a sample collection (PE) bottle at the end of the tube. The PE bottle was removed from the mouth of the net. The 250 ml zooplankton sample was preserved with 1.5 ml of stock Lugol’s Solution. All collected samples were labelled accordingly. The mesh net was rinsed with distilled water after use and was allowed to air-dry after rinsing. The water samples that were set aside for at least 24 hours were decanted leaving only 150 ml of the sample. Quantitative assessment of zooplankton species was adopted from the protocol of Onyema (2007). Here, 1 ml per point per site was obtained after swirling the contents of the remaining solution and was mounted on a glass slide and was covered with a cover slip. In each drop, zooplankton species were counted using the compound light microscope and were identified at the lowest taxonomic level possible using the identification guides (Conway 2012). Since each drop amounts to approximately 0.1 ml, the results on the density of species were multiplied by 10 to represent 1 ml. The zooplankton found were verified in the WoRMS database (WoRMS Editorial Board 2015). Occurrence and abundance (individual l^-1^) of zooplankton were reported in the occurrence.csv dataset. The data are visualised in Fig. 5 and Fig. 6.

**Figure 7.**
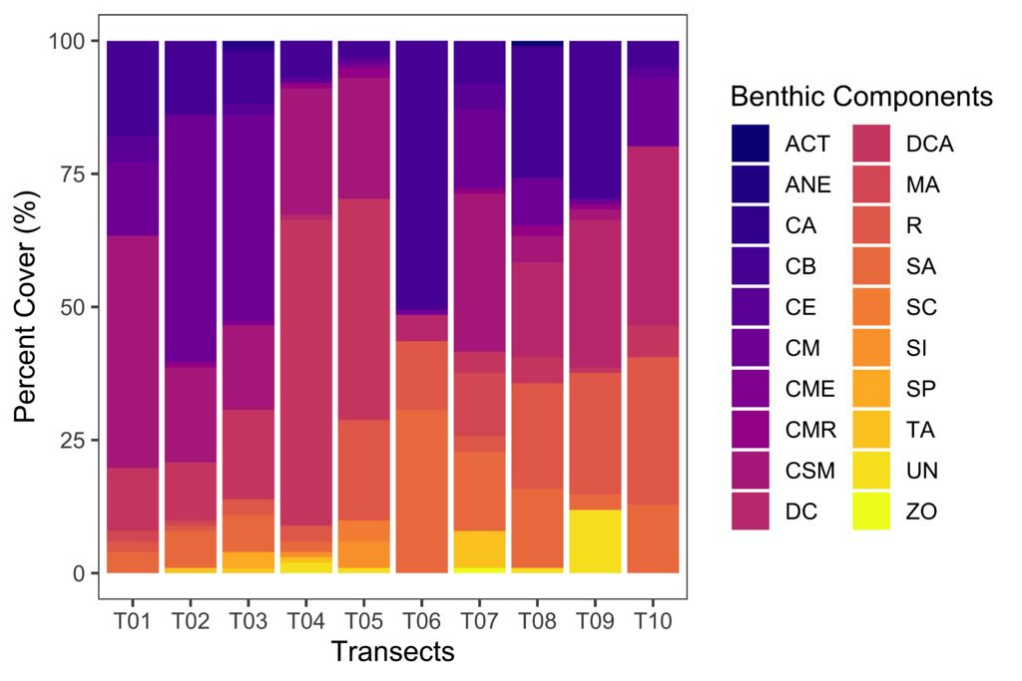
Percent cover (%) of coral reef benthic components including Acropora Table (AT), Anemone (ANE), Coralline Algae (CA), Branching Coral (CB), Encrusting Coral (CE), Massive Coral (CM), Coral Millepora (CME), Coral Mushroom (CMR), Submassive Coral (CSM), Dead Coral (DC), Dead Coral with Algae (DCA), Macroalgae (MA), Rubble (R), Sand (SA), Soft Coral (SC), Silt (SI), Sponge (SP), Turf Algae (TA), Unidentified Abiotic (UN) and Zooanthids (ZO).

**Figure 8.**
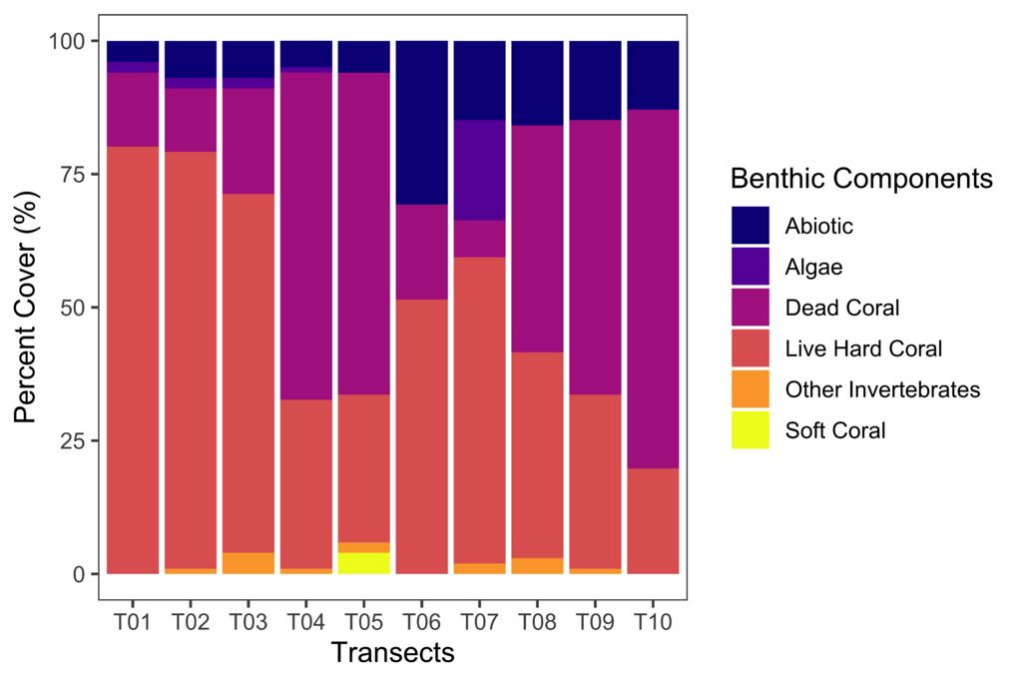
Percent cover (%) of Abiotic (SA, SI, and UN), Algae (MA, CA, and TA), Dead Coral (DC, DCA, R), Live Hard Coral (CB, CM, CSM, CE, CME), other Invertebrates (ACT, ANE, CMR, SP, ZO) and Soft Coral (SC).

**Coral reef benthic composition assessment**: The percent cover of benthic components in Kadurong and Poblacion Reefs was determined by the Point Intercept Transect (PIT) method. Ten 50 m-long transects (T01-T10) were sampled in these reefs. Readings for the benthic life forms were recorded every 0.5 m and a total of 101 points were recorded per transect. The benthic components were characterised using the categories cited in English et al. 1997 and grouped into the following general components: live hard corals, soft corals, dead corals and “others” for other invertebrates and abiotics. The biotic components comprised the live hard corals and soft corals. Live hard corals were specifically categorised into coral morphologies or forms (i.e. branching, massive, sub-massive, encrusting, millepora or fire coral). Dead corals were classified into dead coral with algae, newly-dead coral and rubble. Algae were considered as flora. Non-coralline rocks, sand and silt were herein referred to as abiotic components. Raw data points were collated and summarised into data codes per transects. Each data point identified was given a score of 1 point. All points were then added and divided by the total number of points from all transects and the percentage was taken by multiplying this by 100. The percent cover (%) of each of the components was calculated and reported in the emof.csv dataset. These data are visualised in Fig. 7 and Fig. 8.

**Figure 9.**
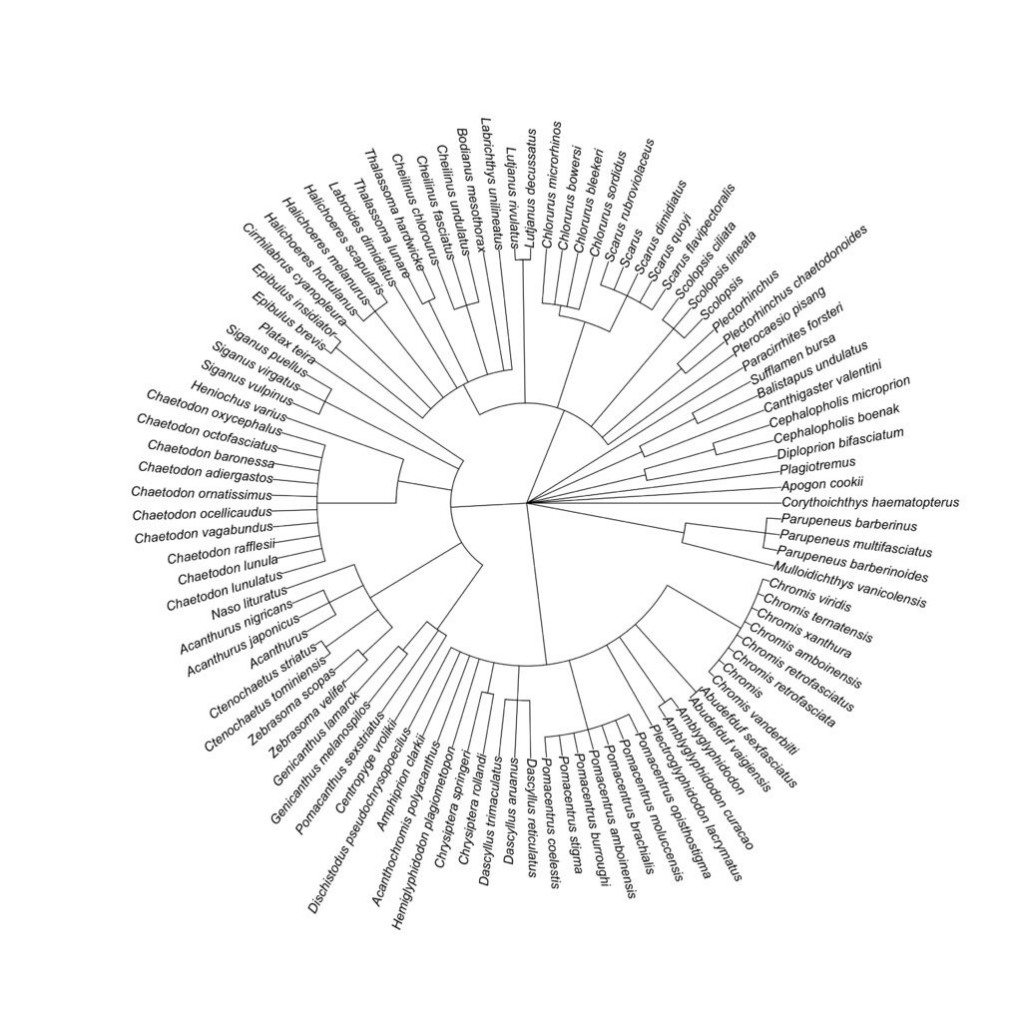
Taxonomic tree of the reef fishes occurring in Kadurong and Poblacion Reefs.

**Figure 10.**
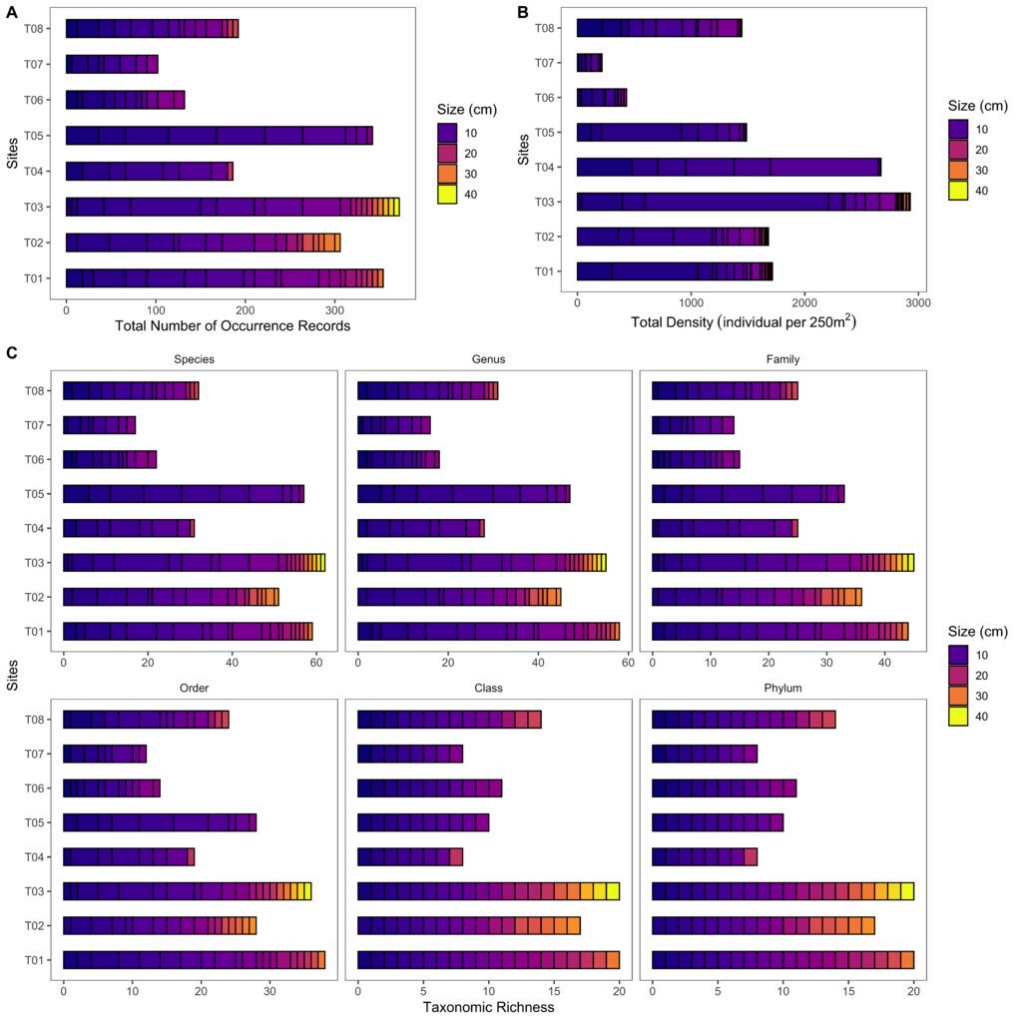
Overview of the fish dataset; **A** total number of occurrence records of fish per class size across sites; **B** total density of fish per size class across sites and **C** total number of species, genus, family, order, class and phylum (taxonomic richness) of fish per class size across sites.

**Reef fish assessment**: The total number of fish families and species were assessed through Underwater Visual Census (UVC) using the same transects (T01-T08) used in PIT. UVC monitoring techniques provide qualitative and quantitative assessments with a limited impact on the ecosystem and are, therefore, particularly suited for marine reserves (Claudet et al. 2006). Divers swam one way along each transect, identifying and recording the number of fish species observed within a distance of 2.5 m on each side of the 50-m transect for 15 minutes. Fishes were identified at the lowest taxonomic level possible. Fish size estimates were also recorded (Samoilys et al. 2007). Fish density per class size (individuals per 250 m^2^) was derived by dividing the total number of individual fish in a 250 m^2^ area. The fishes found were verified in the WoRMS database (WoRMS Editorial Board 2015). Occurrence and density per class size of fishes were reported in the occurrence.csv dataset. These data are visualised in Fig. 9 and Fig. 10.

## Geographic coverage

### Description

The study covered two of the Marine Protected Areas in the Municipality of Liloan, Cebu, Philippines - namely the Poblacion and Kadurong Reefs (Fig. 1).

### Coordinates

10.379° and 10.420° Latitude; 123.984° and 124.036° Longitude.

## Taxonomic coverage

### Description

The study covered occurrences of phytoplankton, zooplankton and fishes in Poblacion and Kadurong Reefs. The occurrence dataset includes 389 occurrences of phytoplankton that belong to four classes, i.e. Bacillariophyceae (42 species), Cyanophyceae (4 species), Dictyochophyceae (1 species) and Dinophyceae (25 species). In addition, the dataset includes observed 94 occurrences of zooplankton that belong to 8 phyla, i.e. Annelida (2 species), Arthropoda (23 species), Bryozoa (1 species), Chaetognatha (1 species), Chordata (1 species), Ciliophora (2 species), Foraminifera (7 species) and Mollusca (2 species). The dataset also includes 331 occurrences of fishes that belong to Class Actinopteri.

### Taxa included

**Table:** 

Rank	Scientific Name	
phylum	Arthropoda	
phylum	Annelida	
phylum	Bryozoa	
phylum	Chaetognatha	
phylum	Chordata	
phylum	Ciliophora	
phylum	Cyanobacteria	
phylum	Foraminifera	
phylum	Mollusca	
phylum	Myzozoa	
phylum	Ochrophyta	

## Temporal coverage

**Formation period:** March 2015; March 2016; July 2016.

### Notes

Coral fish and reef assessments were conducted in March and July 2016 to represent two well-defined climate seasons dictated by the prevailing winds – the northeast monsoon (NE, commonly called “amihan”) and the southwest monsoon (SW, commonly called “habagat”). The NE monsoon prevails from November to early May and is characterised by a dry season with an average precipitation of 75-140 mm. The SW monsoon occurs from May to October and is characterised by hot and humid weather and frequent heavy rainfall (150-200 mm). Physico-chemical and phytoplankton assessments were carried out in March 2015 and 2016 to determine the interannual variability of water quality and trophic status in the NE monsoon. One-time sampling of zooplankton was conducted in March 2015 to determine its community structure during the NE monsoon.

## Usage licence

### Usage licence

Open Data Commons Attribution License

## Data resources

### Data package title

Short-term Monitoring of Coral Reef Marine Protected Areas (MPAs) in the Municipality of Liloan, Central Visayas, Philippines

### Resource link

https://zenodo.org/record/5154878

### Alternative identifiers

https://doi.org/10.5281/zenodo.5154878

### Number of data sets

3

### Data set 1.

#### Data set name

event.csv

#### Data format

Darwin Core Archive (DwC-A)

#### Number of columns

21

#### Character set

UTF-8

#### Download URL

https://zenodo.org/record/5154878/files/event.csv

#### Description

The dataset contains all the necessary details of the sampling events conducted in the Poblaction and Kadurong Reefs.

**Data set 1.** 

Column label	Column description
eventID	An identifier for the set of information associated with an Event.
parentEventID	An identifier for the broader Event that groups this and other Events.
samplingProtocol	The name of, reference to, or description of the method or protocol used during an Event.
year	The four-digit year in which the Event occurred, according to the Common Era Calendar.
month	The integer month in which the Event occurred.
day	The integer day of the month on which the Event occurred.
eventDate	The date-time or interval during which an Event occurred. For occurrences, this is the date-time when the event was recorded. Not suitable for a time in a geological context.
habitat	A category or description of the habitat in which the Event occurred.
eventRemarks	Comments or notes about the Event.
locationID	An identifier for the set of location information (data associated with dcterms:Location). May be a global unique identifier or an identifier specific to the dataset.
country	The name of the country or major administrative unit in which the Location occurs.
countryCode	The standard code for the country in which the Location occurs.
stateProvince	The name of the next smaller administrative region than country (state, province, canton, department, region etc.) in which the Location occurs.
county	The full, unabbreviated name of the next smaller administrative region than stateProvince (county, shire, department etc.) in which the Location occurs.
locality	The specific description of the place. Less specific geographic information can be provided in other geographic terms (higherGeography, continent, country, stateProvince, county, municipality, waterBody, island, islandGroup). This term may contain information modified from the original to correct perceived errors or to standardise the description.
decimalLatitude	The geographic latitude (in decimal degrees, using the spatial reference system given in geodeticDatum) of the geographic centre of a Location. Positive values are north of the Equator, negative values are south of it. Legal values lie between -90 and 90, inclusive.
decimalLongitude	The geographic longitude (in decimal degrees, using the spatial reference system given in geodeticDatum) of the geographic centre of a Location. Positive values are east of the Greenwich Meridian, negative values are west of it. Legal values lie between -180 and 180, inclusive.
geodeticDatum	The ellipsoid, geodetic datum or spatial reference system (SRS), upon which the geographic coordinates given in decimalLatitude and decimalLongitude are based.
coordinateUncertaintyInMetres	The horizontal distance (in metres) from the given decimalLatitude and decimalLongitude describing the smallest circle containing the whole of the Location. Leave the value empty if the uncertainty is unknown, cannot be estimated or is not applicable (because there are no coordinates). Zero is not a valid value for this term.
georeferencedBy	A list (concatenated and separated) of names of people, groups or organisations who determined the georeference (spatial representation) for the Location.
georeferenceProtocol	A description or reference to the methods used to determine the spatial footprint, coordinates and uncertainties.

### Data set 2.

#### Data set name

emof.csv

#### Data format

Darwin Core Archive (DwC-A)

#### Number of columns

9

#### Character set

UTF-8

#### Download URL

https://zenodo.org/record/5154878/files/emof.csv

#### Description

The dataset contains the measurement values of the physico-chemical parameters and coral reef benthic composition in Poblacion and Kadurong Reefs.

**Data set 2.** 

Column label	Column description
eventID	An identifier for the set of information associated with an Event.
measurementID	An identifier for the MeasurementOrFact (information pertaining to measurements, facts, characteristics or assertions). May be a global unique identifier or an identifier specific to the dataset.
measurementType	The nature of the measurement, fact, characteristic or assertion. Recommended best practice is to use a controlled vocabulary.
measurementValue	The value of the measurement, fact, characteristic or assertion.
measurementUnit	The units associated with the measurementValue. Recommended best practice is to use the International System of Units (SI).
measurementDeterminedDate	The date on which the MeasurementOrFact was made. Recommended best practice is to use an encoding scheme, such as ISO 8601:2004(E).
measurementDeterminedBy	A list (concatenated and separated) of names of people, groups or organisations who determined the value of the MeasurementOrFact.
measurementMethod	A description of or reference to (publication, URI) the method or protocol used to determine the measurement, fact, characteristic or assertion.
measurementRemarks	Comments or notes accompanying the MeasurementOrFact.

### Data set 3.

#### Data set name

occurrence.csv

#### Data format

Darwin Core Archive (DwC-A)

#### Number of columns

23

#### Character set

UTF-8

#### Download URL

https://zenodo.org/record/5154878/files/occurrence.csv

#### Description

The dataset contains the phytoplankton, zooplankton and fish occurrence and abundance data in Poblacion and Kadurong Reefs.

**Data set 3.** 

Column label	Column description
eventID	An identifier for the set of information associated with an Event.
occurrenceID	An identifier for the Occurrence (as opposed to a particular digital record of the occurrence). In the absence of a persistent global unique identifier, construct one from a combination of identifiers in the record that will most closely make the occurrenceID globally unique.
basisOfRecord	The specific nature of the data record.
eventDate	The date-time or interval during which an Event occurred. For occurrences, this is the date-time when the event was recorded. Not suitable for a time in a geological context.
scientificName	The full scientific name, with authorship and date information, if known.
taxonRank	The taxonomic rank of the most specific name in the scientificName.
taxonID	An identifier for the set of taxon information (data associated with the taxon class). May be a global unique identifier or an identifier specific to the dataset.
specificEpithet	The name of the first or species epithet of the scientificName.
genus	The full scientific name of the genus in which the taxon is classified.
family	The full scientific name of the family in which the taxon is classified.
order	The full scientific name of the order in which the taxon is classified.
class	The full scientific name of the class in which the taxon is classified.
phylum	The full scientific name of the phylum or division in which the taxon is classified.
kingdom	The full scientific name of the kingdom in which the taxon is classified.
decimalLatitude	The geographic latitude (in decimal degrees, using the spatial reference system given in geodeticDatum) of the geographic centre of a Location. Positive values are north of the Equator, negative values are south of it. Legal values lie between -90 and 90, inclusive.
decimalLongitude	The geographic longitude (in decimal degrees, using the spatial reference system given in geodeticDatum) of the geographic centre of a Location. Positive values are east of the Greenwich Meridian, negative values are west of it. Legal values lie between -180 and 180, inclusive.
geodeticDatum	The ellipsoid, geodetic datum or spatial reference system (SRS), upon which the geographic coordinates given in decimalLatitude and decimalLongitude are based.
coordinateUncertaintyInMetres	The horizontal distance (in metres) from the given decimalLatitude and decimalLongitude describing the smallest circle containing the whole of the Location. Leave the value empty if the uncertainty is unknown, cannot be estimated or is not applicable (because there are no coordinates). Zero is not a valid value for this term.
countryCode	The standard code for the country in which the Location occurs.
individualCount	The number of individuals represented present at the time of the Occurrence.
organismQuantity	A number or enumeration value for the quantity of organisms.
organismQuantityType	The type of quantification system used for the quantity of organisms.
recordedBy	A list (concatenated and separated) of names of people, groups or organisations responsible for recording the original Occurrence. The primary collector or observer, especially one who applies a personal identifier (recordNumber), should be listed first.

## Additional information

R scripts supporting this article are available in Suppl. material 1.

## Supplementary Material

749C0973-0AC6-5B05-9F59-82595F043475 10.3897/BDJ.9.e72537.suppl1 Supplementary material 1 Scripts for exploring the biophysical and biodiversity data Data type R scripts Brief description This contains scripts for exploring the physico-chemical, phytoplankton, zooplankton, benthic composition and fish data described in this paper. File: oo_573968.zip https://binary.pensoft.net/file/573968 Brisneve Edullantes
