# Supplementary figures and images for: A benchmark survey of plankton, fish and benthic composition in Poblacion and Kadurong Reefs in Liloan, Cebu, Philippines
Source: Biodivers Data J. 2021 Sep 23;9:e72537. doi: 10.3897/BDJ.9.e72537 (PMC8484198; doi:10.3897/BDJ.9.e72537)

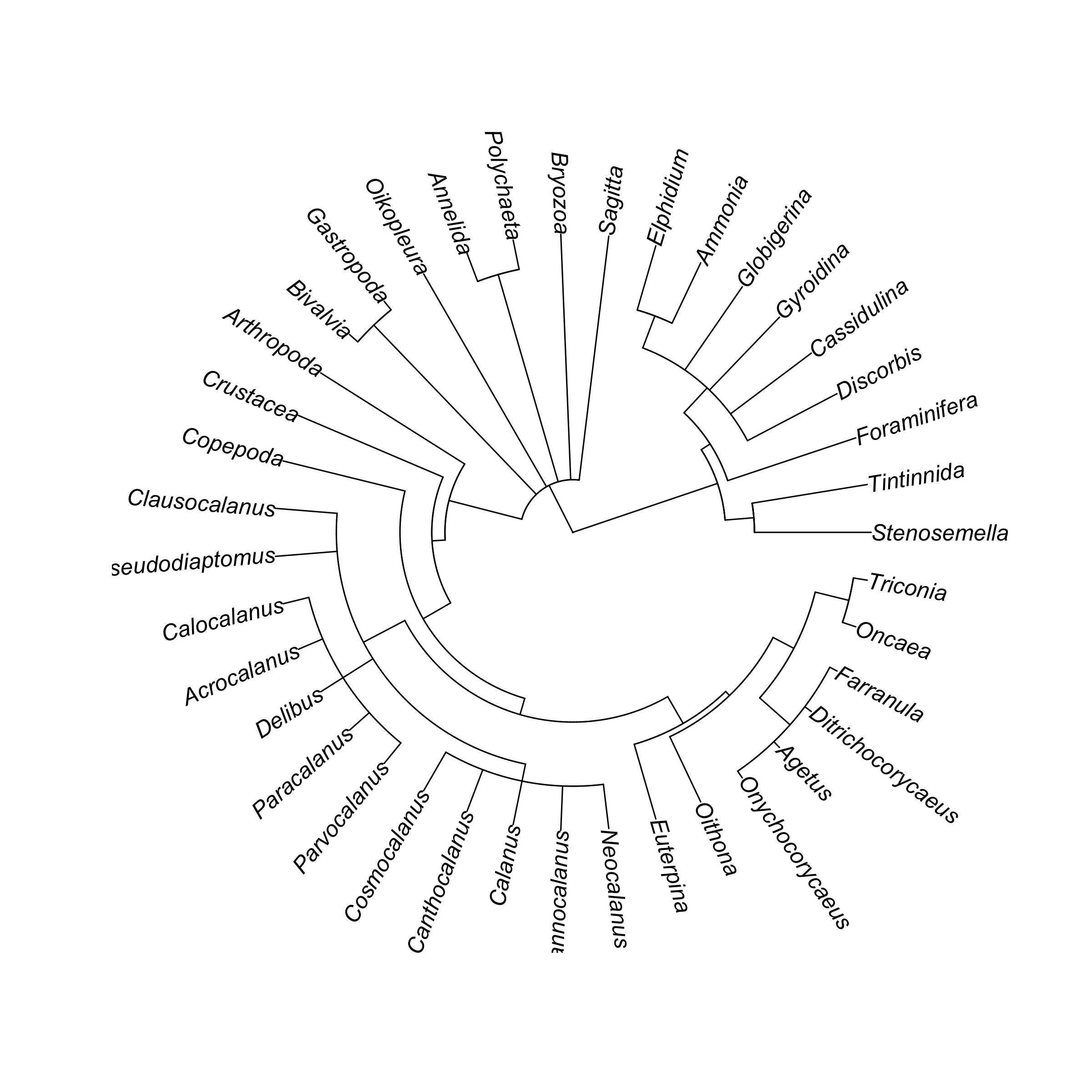

Supplement: Supplementary material 1 — Scripts for exploring the biophysical and biodiversity data [file bdj-09-e72537-s001.zip › JBD-LiloanMPA/Plots/tree_zooplankton.png]

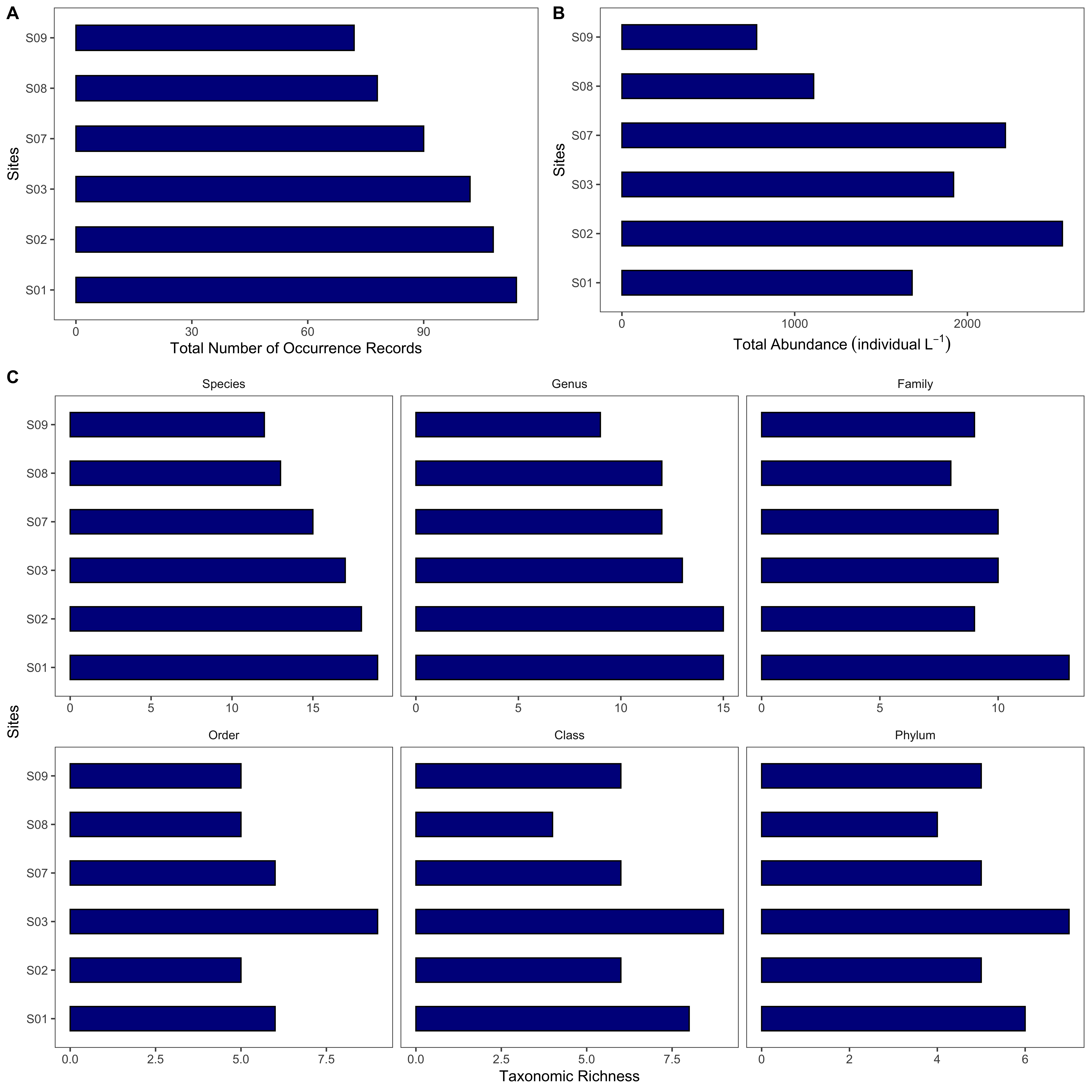

Supplement: Supplementary material 1 — Scripts for exploring the biophysical and biodiversity data [file bdj-09-e72537-s001.zip › JBD-LiloanMPA/Plots/records_zoo.png]

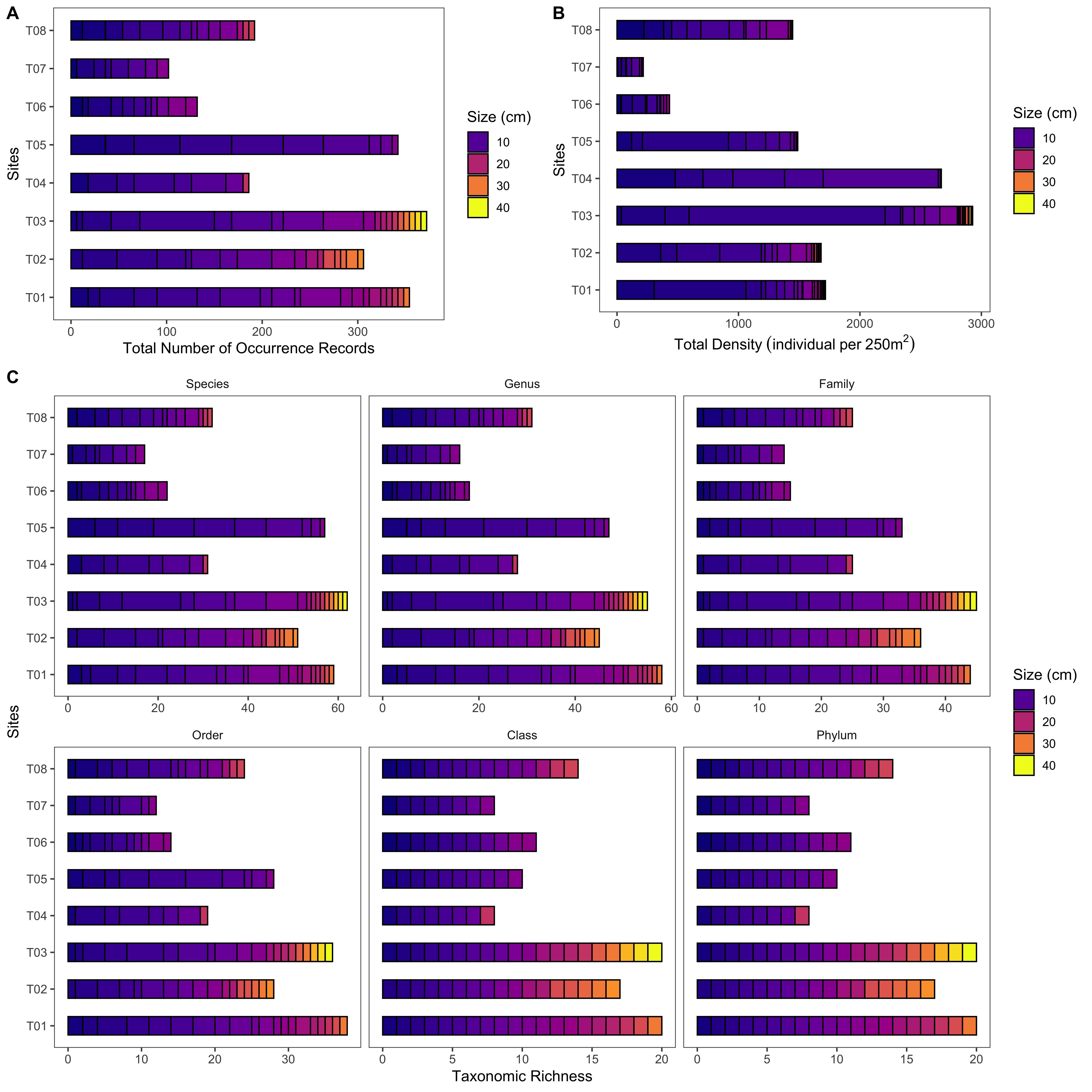

Supplement: Supplementary material 1 — Scripts for exploring the biophysical and biodiversity data [file bdj-09-e72537-s001.zip › JBD-LiloanMPA/Plots/records_fish.png]

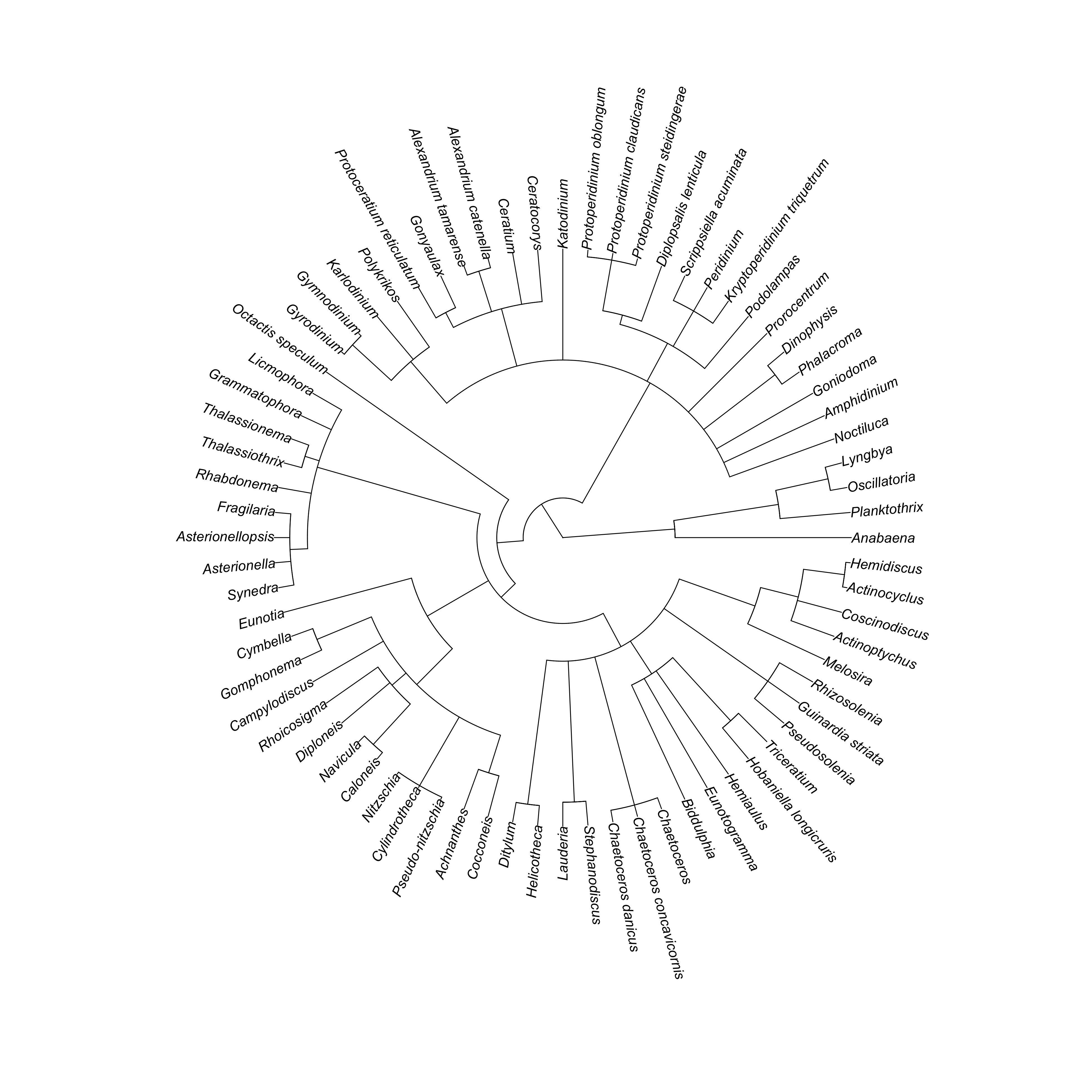

Supplement: Supplementary material 1 — Scripts for exploring the biophysical and biodiversity data [file bdj-09-e72537-s001.zip › JBD-LiloanMPA/Plots/tree_phytoplankton.png]

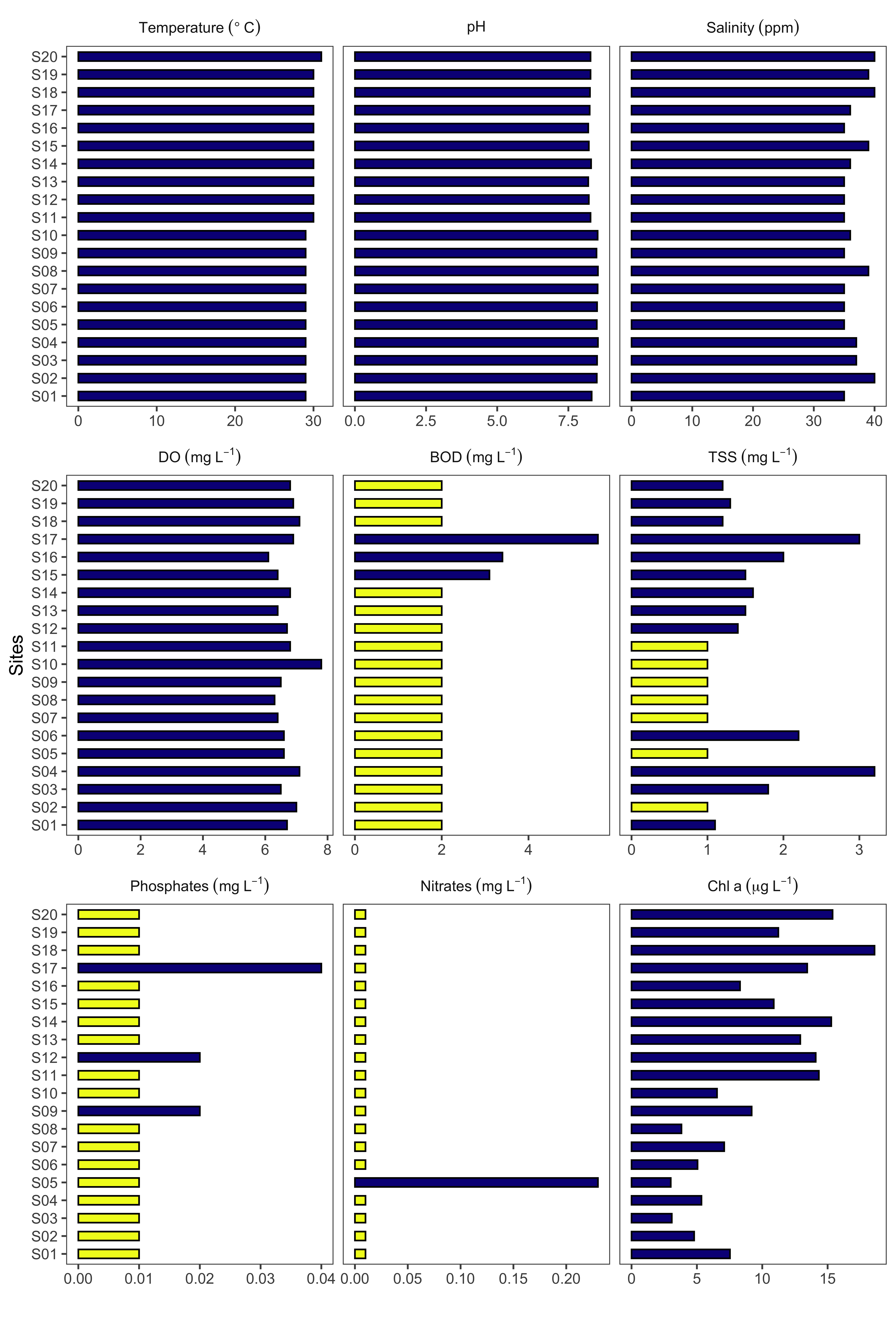

Supplement: Supplementary material 1 — Scripts for exploring the biophysical and biodiversity data [file bdj-09-e72537-s001.zip › JBD-LiloanMPA/Plots/physicochemical.png]

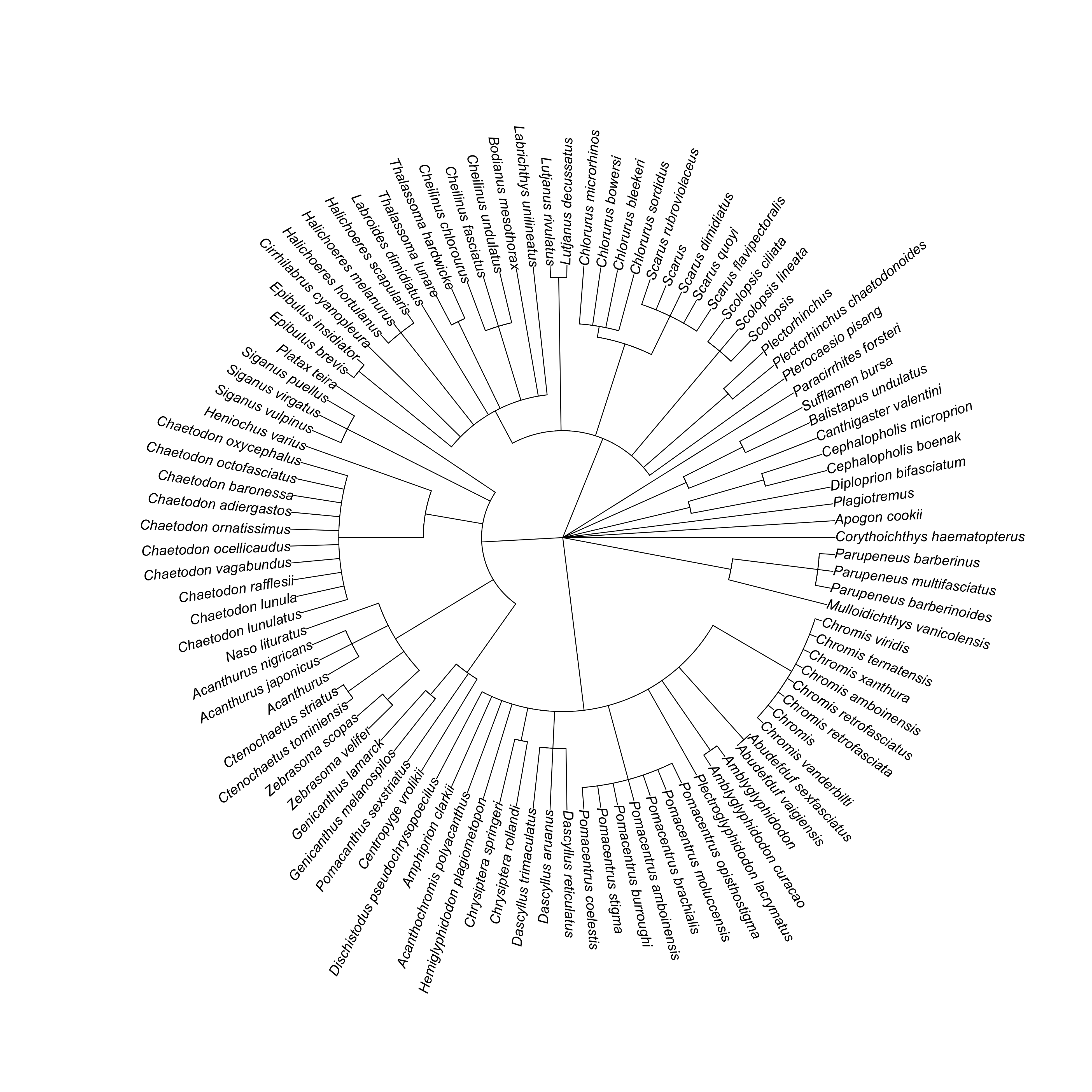

Supplement: Supplementary material 1 — Scripts for exploring the biophysical and biodiversity data [file bdj-09-e72537-s001.zip › JBD-LiloanMPA/Plots/tree_fish.png]

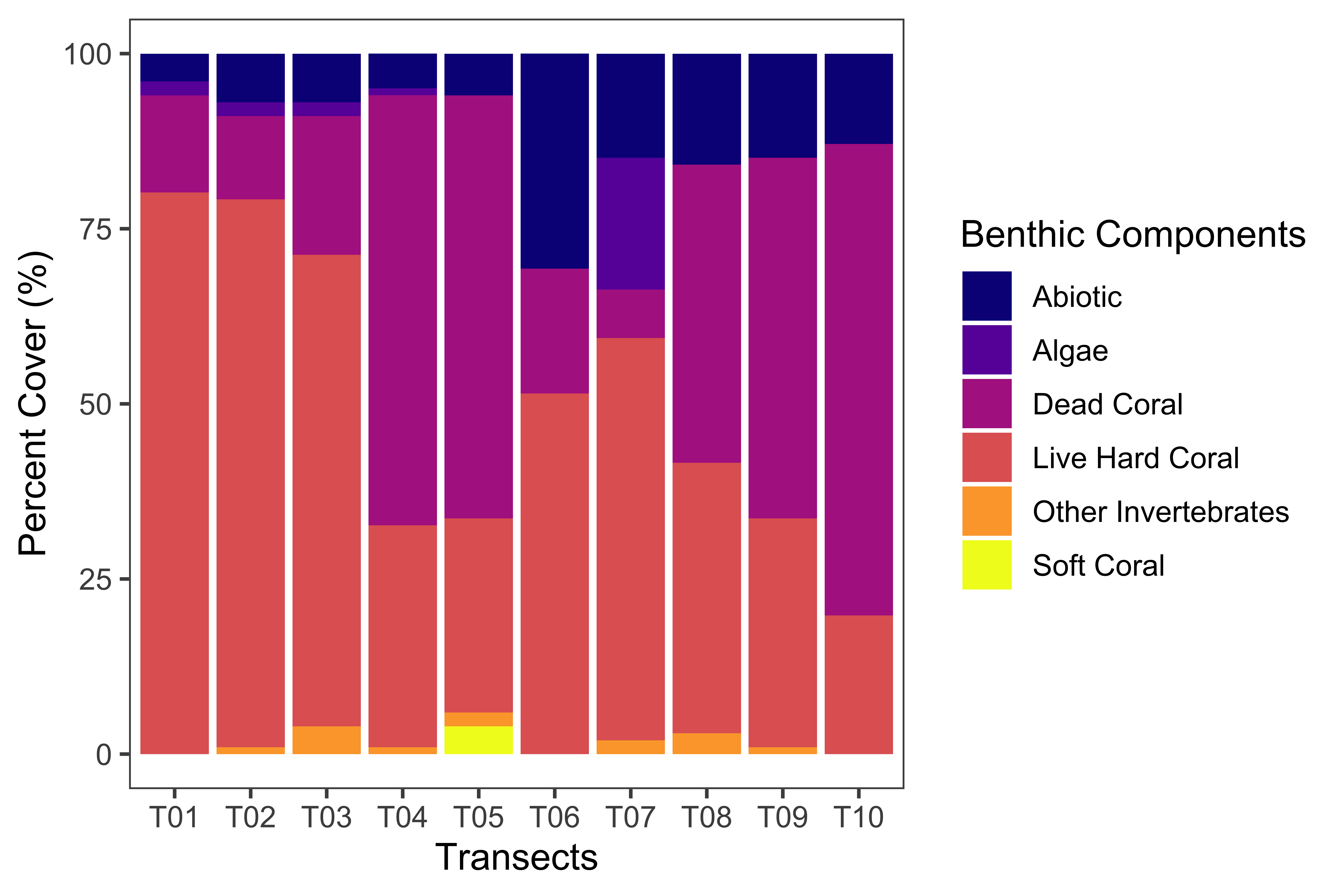

Supplement: Supplementary material 1 — Scripts for exploring the biophysical and biodiversity data [file bdj-09-e72537-s001.zip › JBD-LiloanMPA/Plots/benthic2.png]

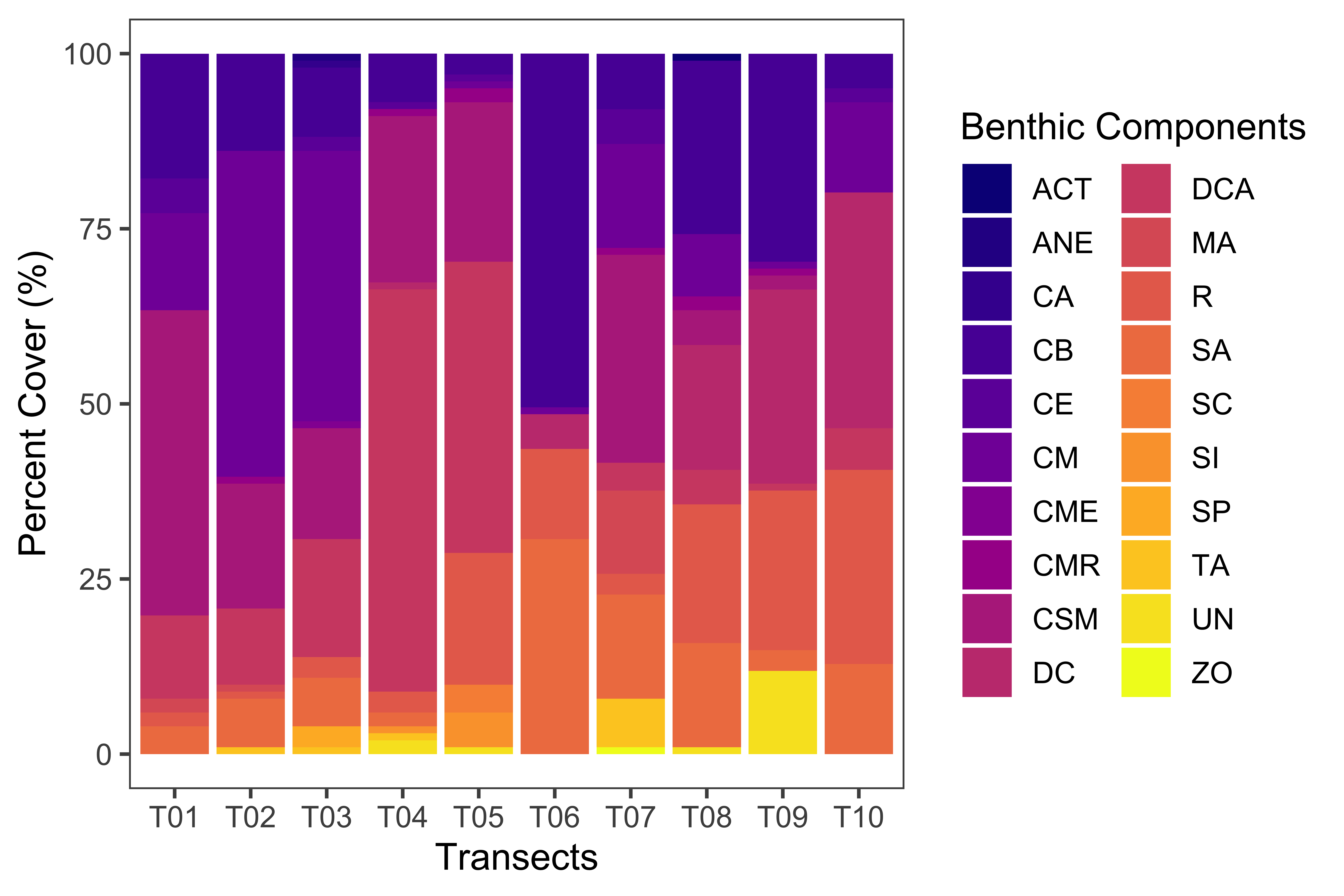

Supplement: Supplementary material 1 — Scripts for exploring the biophysical and biodiversity data [file bdj-09-e72537-s001.zip › JBD-LiloanMPA/Plots/benthic1.png]
